# Synergistic Influence of *Arbuscular mycorrhizal* Fungi Inoculation with Nanoparticle Foliar Application Enhances Chili (*Capsicum annuum* L.) Antioxidant Enzymes, Anatomical Characteristics, and Productivity under Cold-Stress Conditions

**DOI:** 10.3390/plants13040517

**Published:** 2024-02-14

**Authors:** Eman G. Sayed, S. F. Desoukey, Abeer F. Desouky, Mervat F. Farag, Ragab I. EL-kholy, Samah N. Azoz

**Affiliations:** 1 Vegetable Crops Department, Faculty of Agriculture, Cairo University, Giza 12613, Egypt; 2 Agricultural Botany Department, Faculty of Agriculture, Cairo University, Giza 12613, Egyptsamah.ali@agr.cu.edu.eg (S.N.A.); 3 Plant Biotechnology Department, Biotechnology Institute, National Research Center, Dokki, Cairo 12622, Egypt; abeerbiotech@yahoo.com; 4 Horticulture Department, Faculty of Agriculture, Beni Suef University, Beni-Suef 62511, Egypt; mervat.farrag@agr.bsu.edu.eg; 5 Agricultural Botany Department, Genetics Division Faculty of Agriculture, Al-Azhar University, Cairo 11884, Egypt; ragab_elkholy@azhar.edu.eg

**Keywords:** *Capsicum annuum* L., low temperature, *Glomus mosseae*, nano-selenium, nano-zinc oxide

## Abstract

In this study, we aimed to evaluate the effects of *Arbuscular mycorrhiza* fungus (AMF) inoculation, foliar application of zinc oxide and selenium nanoparticles (ZnO-NPs and Se-NPs), and their combined interactions on the growth and productivity of chili pepper under cold-stress conditions. Two field experiments were successfully conducted during the winter seasons of 2021 and 2022 in an experimental field at the Faculty of Agriculture, Cairo University, Giza, Egypt. The results showed that, under cold stress, the combination of AMF inoculation and ZnO-NPs + Se-NPs as a foliar spray increased the average fruit weight by 92.4% and 98.7%, and the number of fruits by 34.6% and 54.8 compared to control treatment in the 2021 and 2022 seasons, respectively. Additionally, the combination of AMF and a mixture of nanoparticles (ZnO-NPs + Se-NPs) significantly increased the total marketable yield by 95.8% and 94.7% compared to the control, which recorded values of 2.4 and 1.9 kg m^−2^ in the 2021 and 2022 seasons, respectively. Furthermore, the combination of AMF and a mixture of nanoparticles (ZnO-NPs + Se-NPs) showed the highest total content of ascorbic acid and capsaicin in chili fruits compared to the other treatments. The combination of AMF and a mixture of nanoparticles (ZnO-NPs + Se-NPs) stimulated the accumulation of peroxidase (POD) and nitrogen glutamate dehydrogenase (GDH) while decreasing hydrogen peroxide (H_2_O_2_) and lipid peroxidation (MDA) contents. SDS analysis revealed that the application of ZnO-NPs, Se-NPs, AMF + ZnO-NPs, and AMF + ZnO-NPs + Se-NPs induced the emergence of new protein bands and reconstitution of those damaged by cold stress. Regarding histological structure, the combination of AMF inoculation and ZnO-NPs + Se-NPs as a foliar spray showed an enhancement in the thickness of grana thylakoids and increased the number of chloroplasts. Intriguingly, the findings showed that AMF and a mixture of nanoparticles (ZnO-NPs + Se-NPs) could offer guidance for increasing plant development and productivity under cold-stress conditions.

## 1. Introduction

Chili (*C. annuum* L.) is commercially grown and consumed for its great nutritional value and spicy flavor [1]. Low temperatures (below 15 °C) have a negative impact on plant growth, flowering, and productivity of chili plants [2]. Additionally, the primary repercussions of cold stress include damage to the chlorophyll content and photosynthesis. Consequently, ROS production damages cell membranes. Plants have resistance systems called osmotic adjustment and antioxidant systems, which protect them from the adverse impacts of cold stress [3]. This enhanced osmolyte and antioxidant formation helps plants resist very low temperatures [4,5]. Furthermore, correlation studies of physiological and biochemical characteristics are very important for managing cold-stress resistance [6]. Hence, plants can adapt to changes in climate by changing their morphological and physiological acclimation [7].

*Arbuscular mycorrhizal* fungus (AMF) inoculation enhances soil structure and regulates nutrient uptake to promote the growth of the host plant [8]. Hence, AMF has been shown to improve plant growth under cold stress [9]. Furthermore, AMF colonization alters the morphological and physiological responses of host plants to stress, as well as increases plant growth and yield [10]. Moreover, AMF inoculation improves root architecture by increasing water and nutrient availability [11]. Additionally, AMF inoculation enhances the rate of photosynthesis by increasing leaf area and phosphorus content [12] and enhancing soil conditions for host plants [13]. The growth of *G. uralensis* plants was improved by inoculation with *Glomus mosseae* and *G. versiforme*, either separately or in combination. Additionally, mycorrhizal growth increases the amount of glycyrrhizic acid in roots [14]. Also, *Glomus. mosseae* and *Glomus. intraradices* can promote glycyrrhizic acid formation in plants of different species. Therefore, AMF inoculation has great potential for enhancing crop yields under field conditions [15]. 

Nanoparticles (NPs) reduce the use of chemical fertilizers and improve the growth of various plant species [16]. Selenium nanoparticles (Se-NPs) enhance root architecture and plant growth [17]. Additionally, Se-NPs alleviate the effects of stress conditions and improve plant growth and hydration [18]. Moreover, the application of selenium (Se) enhances vegetative growth and plant productivity by enhancing the antioxidant defense system under abiotic stress [19]. Foliar spray treatment, as opposed to soil supplementation, is an efficient alternative method for boosting Se levels in crops. Foliar nourishment with Se reduces the effects of soil chemistry, allowing for efficient absorption even at trace levels [20]. Thus, strawberry seedlings sprayed with Se have a higher photosynthetic rate under cold stress [20]. Zinc oxide nanoparticles (ZnO-NPs) are used worldwide because of their positive environmental impacts [21]. ZnO-NPs have exceptional photooxidation and photocatalytic abilities against chemical and biological species [22]. Furthermore, ZnO-NPs are crucial for both physiological and anatomical processes [23]. Hence, ZnO-NPs can improve the productivity of several vegetable crops [24]. Moreover, SDS proteins are useful in offering reactions for genetic conservation, where there is a need to assess genetic variability in plant species [25].

Based on the results of this study, we aimed to determine whether the combined effects of AMF inoculation and foliar application of Se-NPs and ZnO-NPs are more effective than their individual effects in improving chili plant growth and productivity under cold-stress conditions.

## 2. Results

### 2.1. Plant Growth and Flowering Traits of Chili Plants

**Figure 1:**
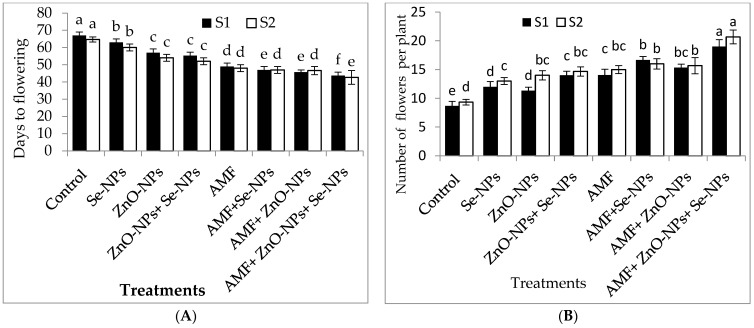
Effect of inoculation with *Arbuscular mycorrhiza* fungus (AMF), foliar applications of ZnO-NPs, foliar applications of Se-NPs, ZnO-NPs + Se-NPs, AMF + ZnO-NPs, AMF + Se-NPs, AMF + ZnO-NPs + Se-NPs, and untreated plants (control) on (**A**) days to flowering, and (**B**) number of flowers of chili plants during (S1: 2021 and S2: 2022) seasons. Vertical bars indicate the standard error of the mean. The LSD test demonstrates that differences among values in each bar that are marked by different letters (a–f) are significant at *p* ≤ 0.05.

As shown in Table 1, all treatments exhibited significantly (*p* ≤ 0.05) increased plant growth characteristics compared to the control in both seasons. The greatest plant height, number of leaves, plant fresh weight, and plant dry weight were recorded for AMF inoculation combined with a mixture of nanoparticles (ZnO-NPs + Se-NPs) as a foliar spray in both seasons. Moreover, the leaf area increased by 54.8% and 50.5% following AMF inoculation combined with a nanoparticle mixture (ZnO-NPs + Se-NPs) compared to the control plants in 2021 and 2022, respectively. The flowering time and number of flowers were significantly influenced by all tested treatments in both seasons (Figure 1). Chili plants inoculated with AMF combined with the nanoparticle mixture (ZnO-NPs + Se-NPs) had the highest number of flowers compared to the control plants in both seasons. Furthermore, chili plants inoculated with AMF combined with a nanoparticle mixture (ZnO-NPs + -NPs) flowered earlier than control plants by between 23 and 22 days in the 2021 and 2022 seasons, respectively.

### 2.2. Photosynthetic Pigments of Chili Plants 

**Figure 2:**
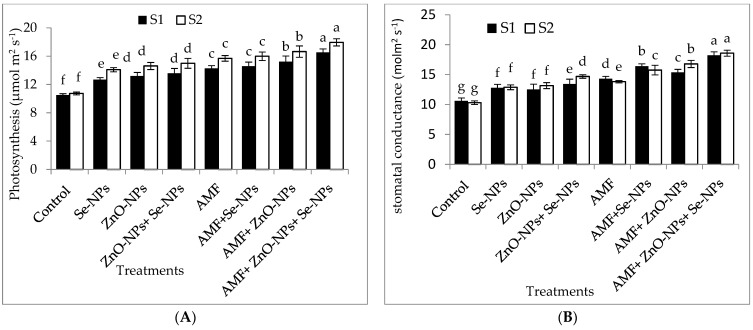

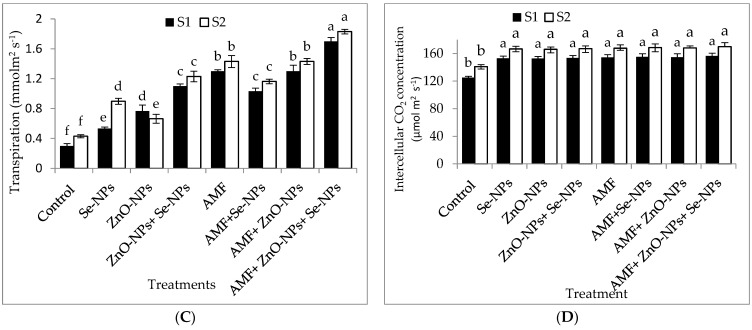
Effect of inoculation with *Arbuscular mycorrhiza* fungus (AMF), foliar applications of ZnO-NPs, foliar applications of Se-NPs, ZnO-NPs + Se-NPs, AMF + ZnO-NPs, AMF + Se-NPs, AMF + ZnO-NPs + Se-NPs, and untreated plants (control) on (**A**) photosynthesis rate, (**B**) stomatal conductance, (**C**) transpiration rate, and (**D**) intercellular CO_2_ concentration of chili plants during (S1: 2021 and S2: 2022) seasons. Vertical bars indicate the standard error of the mean. The LSD test demonstrates that differences among values in each bar that are marked by different letters (a–f) are significant at *p* ≤ 0.05.

The data in Figure 2A–D clearly show that AMF inoculation and the foliar application of ZnO-NPs with or without + Se-NPs significantly (*p* ≤ 0.05) affected the relative chlorophyll content (SPAD) and photosynthetic measurements compared to the control in both seasons. Chili plants inoculated with AMF combined with foliar application and a mixture of nanoparticles (ZnO-NPs + Se-NPs) exhibited better transpiration rate, stomatal conductance, and photosynthetic rate than the control plants in the 2021 and 2022 seasons. Moreover, the relative chlorophyll content significantly increased by 55.13% and 60.7% following AMF inoculation in combination with the nanoparticle mixture (ZnO-NPs + Se-NPs) as a foliar spray compared to the control chili plants during the 2021 and 2022 seasons, respectively. Likewise, the combination of inoculation with AMF and the mixture of nanoparticles (ZnO-NPs + Se-NPs) significantly increased the photosynthesis rate by 54.5% and 63.6% compared with the control plants in the 2021 and 2022 seasons, respectively. Furthermore, there was a significant increase in intercellular CO_2_ concentrations in response to treatment with either Se-NPs or ZnO-NPs foliar application in the absence or presence of AMF on chili plants, without any significant difference between them, compared to the control in both seasons. In contrast, the control chili plants had the lowest intercellular CO_2_ concentrations during both seasons. 

### 2.3. Marketable Yield and Its Components

**Figure 3:**
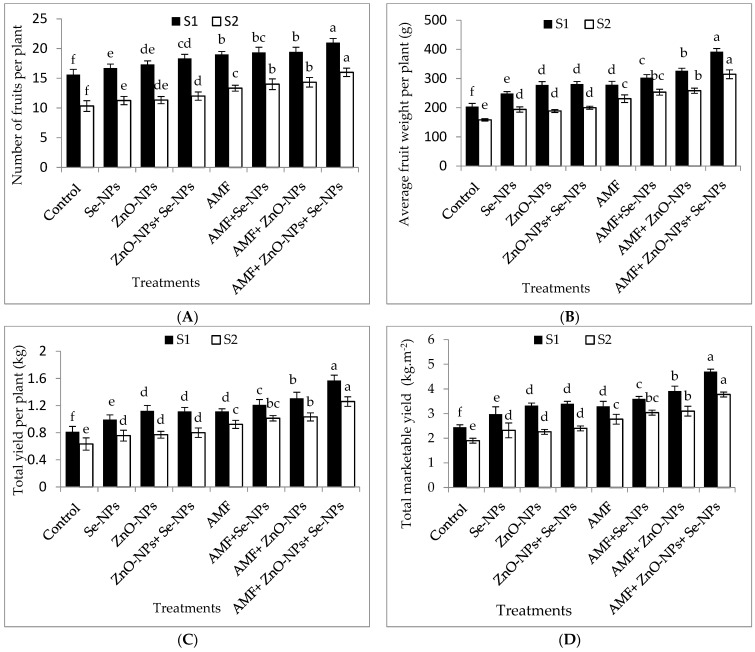
Effect of inoculation with *Arbuscular mycorrhiza* fungus (AMF), foliar applications of ZnO-NPs, foliar applications of Se-NPs, ZnO-NPs + Se-NPs, AMF + ZnO-NPs, AMF + Se-NPs, AMF + ZnO-NPs + Se-NPs, and untreated plants (control) on (**A**) number of fruits per plant, (**B**) average fruit weight per plant, (**C**) total yield per plant, and (**D**) total marketable yield per (m^2^) of chili plants during (S1: 2021 and S2: 2022) seasons. Vertical bars indicate the standard error of the mean. The LSD test demonstrates that differences among values in each bar that are marked by different letters (a–f) are significant at *p* ≤ 0.05.

The results in Figure 3A–D clearly illustrate that chili pepper plants inoculated with AMF significantly (*p* ≤ 0.05) influenced the number of fruits per plant, average fruit weight, total fruit yield per plant, and total marketable fruit yield per m^2^ compared to the control plants in both seasons. Likewise, foliar spraying of ZnO-NPs with or without Se-NPs drastically (*p* ≤ 0.05) enhanced the marketable yield compared to the control chili plants in both seasons. Chili plants inoculated with AMF and sprayed with (ZnO-NPs + Se-NPs) significantly increased average fruit weight per plant by (92.4% and 98.7%), number of fruits per plant by (34.6% and 54.8%), and total yield per plant by (93.8% and 90.4%) compared to the control in 2021 and 2022 seasons, respectively. Hence, the marketable yields of pepper plants recorded the highest values (4.7 and 3.8 kg fruits m^−2^) when plants inoculated with AMF in combination with nanoparticles (ZnO-NPs + Se-NPs), which significantly increased by 95.8% and 94.7% in 2021 and 2022, respectively, compared to the control plants. Nevertheless, the control plants recorded the lowest number of fruits per plant, average fruit weight, total fruit yield per plant, and marketable yield during both seasons.

### 2.4. Quality Parameters of Chili Fruits

Chili fruit quality traits were significantly (*p* ≤ 0.05) affected by AMF inoculation, nanoparticles foliar application, and their combined interactions (Table 2). Fruit quality parameters, such as fruit length, single-fruit weight, fruit diameter, and capsaicin content, displayed significant variation between the tested treatments and control plants in both seasons (Table 2). The highest weight and length of fruits were recorded following the inoculation of AMF combined with the nanoparticle mixture ZnO-NPs and Se-NPs as a foliar application in the 2021 and 2022 seasons, respectively. Additionally, the inoculation of AMF with foliar spray of ZnO-NPs and Se-NPs drastically increased the average fruit weight by 43.6% and 28.3% compared to the control in the 2021 and 2022 seasons, respectively. Furthermore, AMF combined with ZnO-NPs with or without Se-NPs recorded the highest fruit diameter compared to the control in both years of the experiment. Additionally, harvested chili fruits from all treatments, except for the plants sprayed with ZnO-NPs, showed higher levels of ascorbic acid than the control in both seasons (Figure 3B). Moreover, the highest ascorbic acid content (125 and 129 mg/100 g FW) was recorded in chili fruits treated with AMF combined with nanoparticles mixture (ZnO-NPs + Se-NPs) foliar application, which significantly increased by 46.1% and 46.6%in the 2021 and 2022 seasons, respectively. Additionally, the highest content of capsaicin (142.9 and 143.3 mg/100 g FW) was recorded in chili fruits treated with AMF combined with nanoparticles mixture (ZnO-NPs + Se-NPs) foliar application, which significantly increased by 7.2 and 6.9% in 2021 and 2022 seasons, respectively, compared with untreated plants.

### 2.5. Mineral Content in Chili Leaves 

The results in Table 3 show that all tested treatments had a significant (*p* ≤ 0.05) improvement in macronutrient concentrations, such as nitrogen (N), phosphorus (P), potassium (K), and calcium (Ca), in the leaves of chili plants compared with the control. The combination of AMF with a mixture of (ZnO-NPs + Se-NPs) increased the concentration of N in leaves by 56% and 82.6% compared to the control during the 2021 and 2022 seasons, respectively. In addition, the combination of AMF with a mixture of ZnO-NPs and Se-NPs resulted in maximum concentrations of P, K, and Ca compared to the control treatment. In contrast, control plants showed the lowest concentrations of N, P, K, and Ca during the 2021 and 2022 seasons. Concerning the effect of treatments on the concentration of micronutrients in chili leaves (Table 4), selenium (Se), zinc (Zn), iron (Fe), copper (Cu), and manganese (Mn) were considerably influenced by AMF inoculation, foliar spraying of nanoparticles, and their combined interactions in both seasons. Moreover, the combination of AMF with a mixture of ZnO-NPs and Se-NPs exhibited the highest concentrations of Se, followed by AMF + Se-NPs in the 2021 and 2022 seasons, respectively, compared to the control treatment. Furthermore, the combination of AMF with a mixture of ZnO-NPs and Se-NPs showed the highest concentrations of Zn, followed by AMF + ZnO-NPs and ZnO-NPs + Se-NPs in the 2021 and 2022 seasons, respectively, compared to the control treatment. Additionally, the highest concentrations of Mn, Cu, and Fe were found in chili plants treated with AMF and combined nanoparticles (ZnO-NPs + Se-NPs) compared to the control in 2021 and 2022, respectively. On the other hand, the control plants recorded the lowest concentrations of Se, Zn, Mn, Cu, and Fe during the 2021 and 2022 seasons.

### 2.6. Plant Hormones, Nitrogen Metabolism Enzyme, Antioxidant Enzymes, Hydrogen Peroxide, and Lipid Peroxidation Content in Chili Leaves

**Figure 4:**
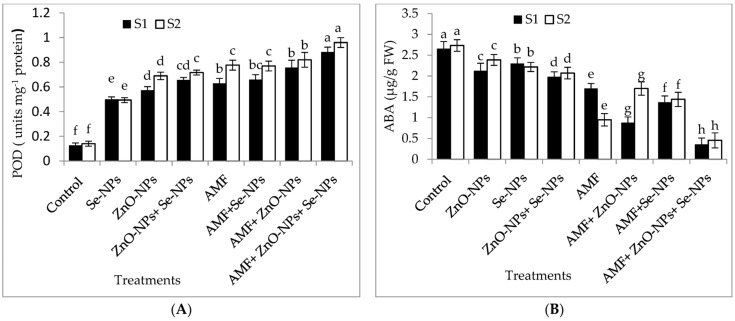

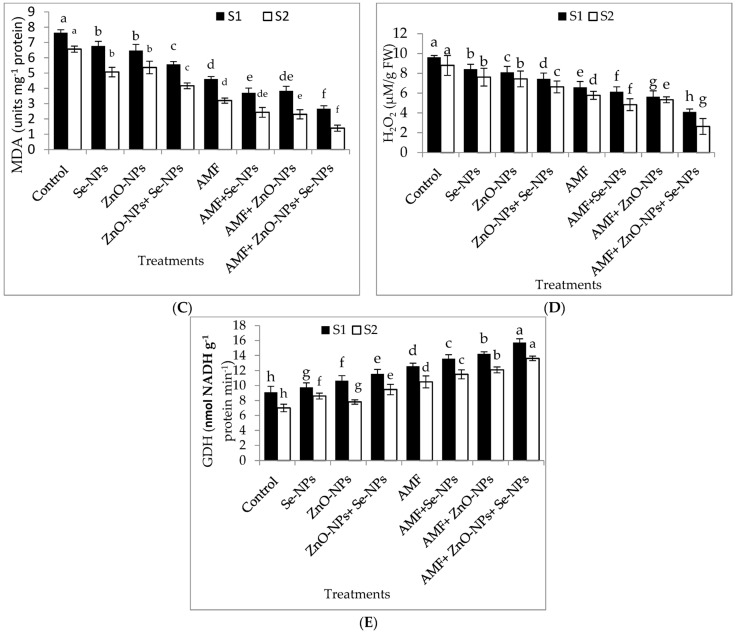
Effect of inoculation with *Arbuscular mycorrhiza* fungus (AMF), foliar application of ZnO-NPs, foliar application of Se-NPs, ZnO-NPs + Se-NPs, AMF + ZnO-NPs, AMF + Se-NPs, AMF + ZnO-NPs + Se-NPs, and untreated plants (control) on (**A**) peroxidase (POD), (**B**) abscisic acid (ABA), (**C**) malondialdehyde (MDA), (**D**) hydrogen peroxide (H_2_O_2_), and (**E**) glutamate dehydrogenase (GDH) of chili leaves during (S1: 2021 and S2: 2022) seasons. Vertical bars indicate the standard error of the mean. The LSD test demonstrates that differences among values in each bar that are marked by different letters (a–h) are significant at *p* ≤ 0.05.

The results in Figure 4A–E demonstrate the effects of AMF inoculation, nanoparticles foliar application, and their combined interactions on the content of the most crucial hormones, abscisic acid (ABA) and peroxidase (POD) antioxidant enzyme, and malondialdehyde (MDA), hydrogen peroxide (H_2_O_2_), and glutamate dehydrogenase (GDH) nitrogen metabolism enzyme. The combination of AMF with nanoparticles mixture (ZnO-NPs + Se-NPs) exhibited better POD activity than control in the 2021 and 2022 seasons. In contrast, the control plants registered the lowest POD content in both seasons. Furthermore, the combination of AMF with nanoparticles mixture (ZnO-NPs + Se-NPs) decreased the content of ABA by (86.45% and 83.3%) in chili leaves compared to the control in the 2021 and 2022 seasons, respectively. In contrast, the control chili plants had the highest ABA concentrations during both seasons. Correspondingly, AMF combined with a nanoparticle mixture (ZnO-NPs + Se-NPs) mitigated the adverse impact of cold stress on the activity of the nitrogen metabolism enzyme, with the activity of GDH increasing by 72.8% and 94.7%, respectively, compared with control plants in the 2021 and 2022 seasons, respectively (Figure 4E). By contrast, AMF combined with a nanoparticle mixture (ZnO-NPs + Se-NPs) decreased the content of MDA by (65.7% and 78.4%) and the content of H_2_O_2_ by (70.4% and 78.6%) in chili leaves compared to the control in the 2021 and 2022 seasons, respectively. However, untreated plants had the highest MDA and H_2_O_2_ contents in the leaves in both seasons.

### 2.7. Protein Electrophoresis SDS-PAGE

**Figure 5:**
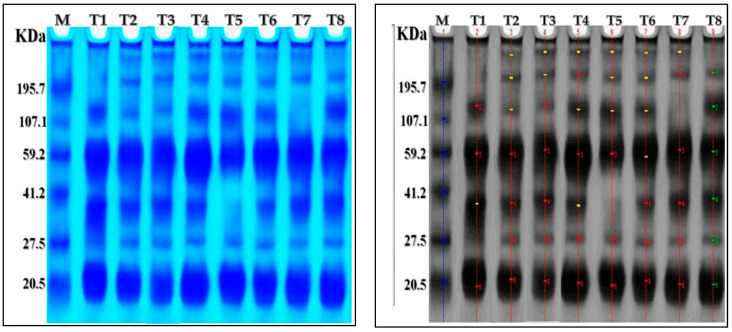
The change in protein bands (marked by arrowheads) in response to tested treatments on pepper plants under cold stress. Electrograph of soluble protein pattern by one-dimensional SDS-PAGE. As shown, lane one is a molecular weight marker with six bands of known size ranging from 20.74 to 320.23 KDA. T1: lane is control treatment, T2: is Se-NPs, T3: ZnO-NPs, T4: AMF + ZnO-NPs + Se-NPs, T5: AMF + Se-NPs, T6: AMF + ZnO-NPs, T7: AMF, T8: ZnO-NPs + Se-NPs.

AMF inoculation, nanoparticles foliar application, and their combined interactions stimulated the appearance or disappearance of different protein bands compared to control plants (Table 5 and Figure 5). There were seven protein bands ranging from 20.74 to 320.23 kDa in the chili pepper protein patterns. The control plants showed four bands with molecular weights of 133.56, 61.96, 38.82, and 20.74 kDa. Furthermore, ZnO-NPs, Se-NPs, AMF + ZnO-NPs + Se-NPs, and AMF + ZnO-NPs induced the synthesis of new bands with molecular weights of 320.23, 228, and 38.2 kDa. The treatment of AMF + Se-NPs led to the disappearance of 38.2 compared to the control plants. The treatment with AMF led to the disappearance of the 133.56 kDa compared to the control. Thus, we concluded that cold stress affected expression in the control, as the disappearance of three bands at various molecular weights appeared in different treatments, as ZnO-NPs, Se-NPs, AMF + ZnO-NPs + Se-NPs, and AMF + ZnO-NPs provided plants with a defense mechanism against cold stress compared with the control.

### 2.8. Leaf Anatomy

**Figure 6:**
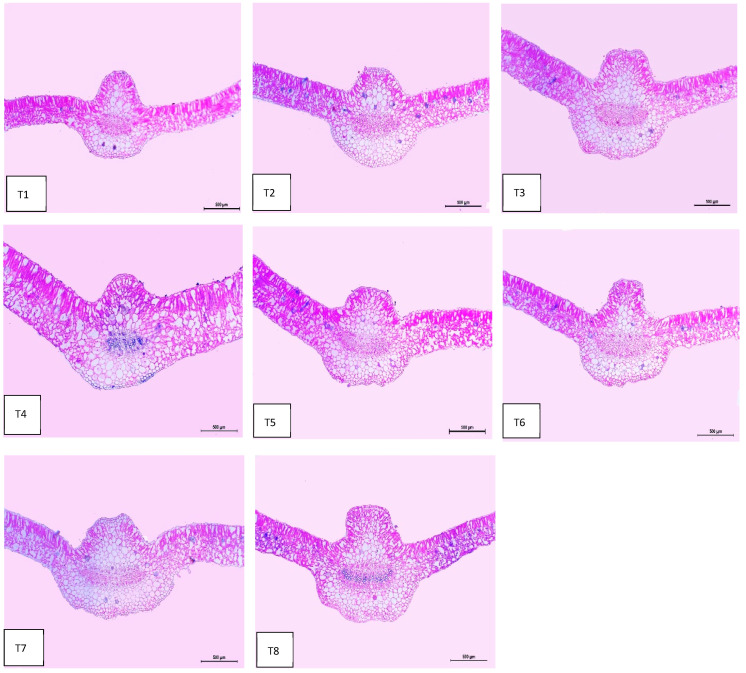
Microphotographs of cross sections through the chili pepper leaf, treated with *Arbuscular mycorrhiza* fungus (AMF) and foliar application of nanoparticles after 45 days from transplanting. Scale bars = 500 µm. (**T1**) control (untreated plants); (**T2**) Se-NPs; (**T3**) ZnO-NPs; (**T4**) ZnO-NPs + Se-NPs; (**T5**) AMF inoculation; (**T6**) AMF + Se-NPs; (**T7**) AMF + ZnO-NPs and (**T8**) AMF + (ZnO-NPs + Se-NPs).

**Figure 7:**
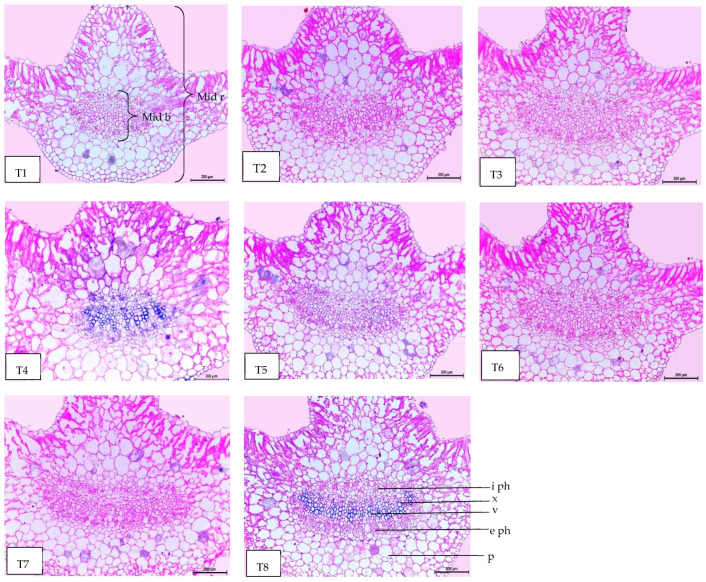
Magnified portions of the leaf midvein bundle of chili pepper plants after 45 days from transplanting. Scale bars = 200 µm. (**T1**) control (untreated plants); (**T2**) Se-NPs; (**T3**) ZnO-NPs; (**T4**) ZnO-NPs + Se-NPs; (**T5**) AMF inoculation; (**T6**) AMF + Se-NPs; (**T7**) AMF + ZnO-NPs and (**T8**) AMF + (ZnO-NPs + Se-NPs). Details: mid b, midvein bundle; mid r, midvein region; e ph, external phloem; i ph, internal phloem; v, vessel; x, xylem and p, parenchyma.

**Figure 8:**
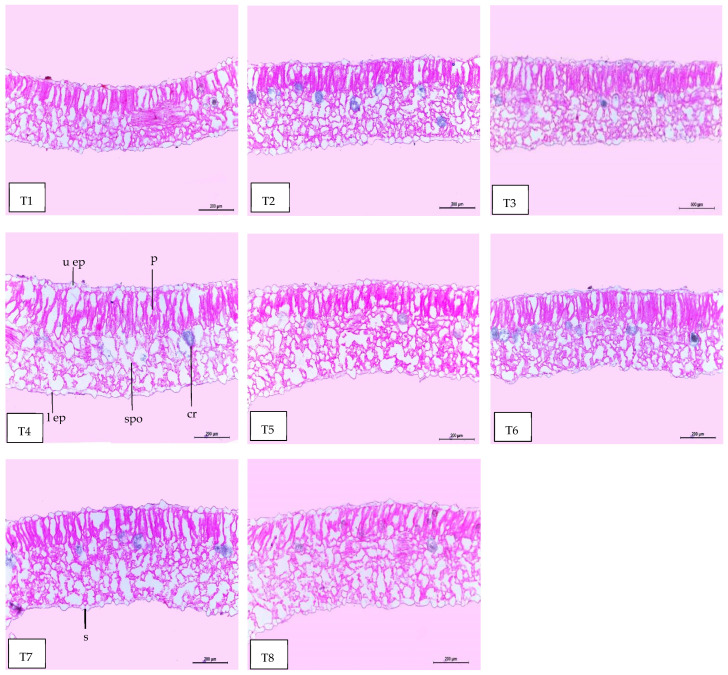
Magnified portions of the leaf blade lamina of chili pepper plants after 45 days from transplanting. Scale bars = 200 µm. (**T1**) control (untreated plants); (**T2**) Se-NPs; (**T3**) ZnO-NPs; (**T4**) ZnO-NPs + Se-NPs; (**T5**) AMF inoculation; (**T6**) AMF + Se-NPs; (**T7**) AMF + ZnO-NPs and (**T8**) AMF + (ZnO-NPs + Se-NPs). Details: l ep, lower epidermis; u ep, upper epidermis; cr, cells with dark content containing calcium oxalate crystals; s, stomata; spo, spongy tissue, and p, palisade tissue.

Regarding anatomical maintenance, chili leaves have adaxial and abaxial epidermis with stomata on both sides. The epidermal cells were covered with a slim cuticle layer on the surface of the outer walls. The mesophyll was composed of 3–5 layers of spongy parenchyma cells with intercellular gaps and one layer of palisade parenchyma cells. In the midrib, the vascular bundle was bicollateral, with the phloem facing both sides of the leaf and surrounded by collenchyma cells Figure 6, Figure 7 and Figure 8. As shown in Table 6 and Figure 6, Figure 7 and Figure 8, the upper epidermis, thickness of the midvein, and lower epidermis of the chili plants inoculated with AMF combined with (ZnO-NPs + Se-NPs) increased by 20.41%, 34.15%, and 13.99%, respectively. Correspondingly, the thickness of the vascular bundle of the midvein was enhanced owing to the increase in the dimensions of the main midvein bundle and the mean diameter of the vessels by 67.72% and 12.84%, respectively, when compared with the control (Figure 6, Figure 7 and Figure 8, T1 and T8). A promotive effect on leaf lamina thickness was observed in plants sprayed with ZnO-NPs + Se-NPs by 65.85%, owing to an enhancement in the thickness of palisade and spongy tissues by 62.0% and 68.15%, respectively, when compared with the control (Figure 8, T1 and T4). A minimal improvement was observed at Se-NPs in the thickness of the midvein, palisade, lamina, and spongy tissues by 18.16%, 4.47%, 10.84%, and 15.22%, respectively, compared to the control. Additionally, the upper and lower epidermis thickness increased by 0.44% and 3.43%, respectively, compared to the control (Figure 6, Figure 7 and Figure 8, T1 and T2).

### 2.9. Mesophyll Parenchyma and Chloroplast Ultrastructure of Chili Pepper Leaves

**Figure 9:**
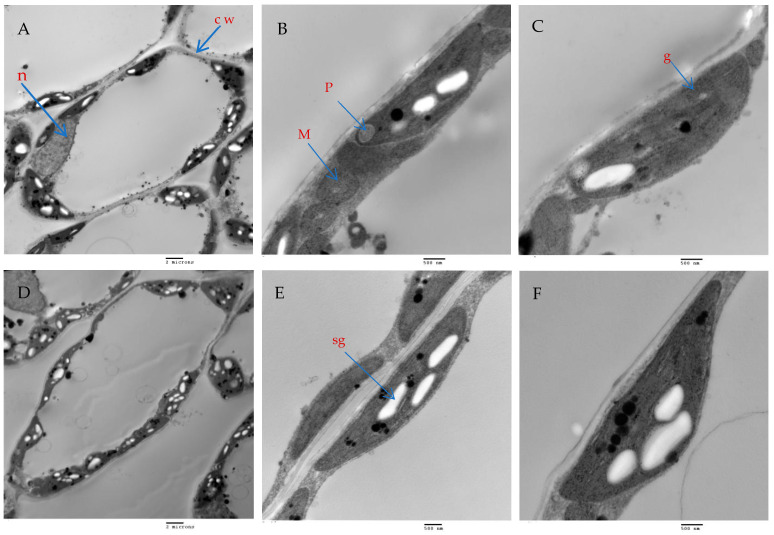
The effect of AMF + (ZnO-NPs + Se-NPs) on mesophyll parenchyma and chloroplast ultrastructure in pepper leaves: (**A**–**C**) Control plants, (**D**–**F**) Plants treated with AMF + (ZnO-NPs + Se-NPs). Details: cw, cell wall; n, nucleus; sg, starch granule; p, plastoglobule; m, mitochondria and g, grana. (**A**,**D**) scale bars = 2 m; (**B**,**E**) scale bars 500 nm; (**C**,**F**): scale bars = 500 nm.

**Figure 10:**
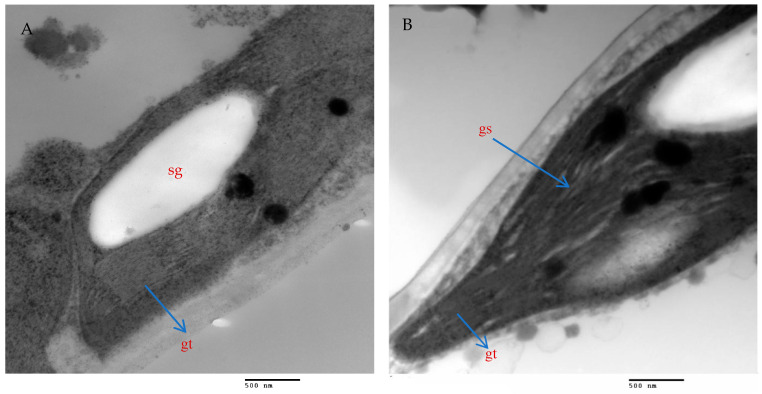
Magnified portions of the chloroplast ultrastructure in chili leaves. Scale bars = 500 nm. (**A**)—Control plants; (**B**)—Plants treated with AMF + (ZnO-NPs + Se-NPs). Details: sg, starch granule; gt, stacked grana thylakoids; gs, non-stacked stroma thylakoids.

The mesophyll parenchyma of the chili pepper leaves displayed significant differences between the tested treatments. The mesophyll cells of the chili plants treated with AMF with a mixture of nanoparticles (ZnO-NPs + Se-NPs) were larger than those of the control plants (Figure 9A,D). The chloroplasts in the mesophyll cells of the chili plants inoculated with AMF and sprayed with a mixture of nanoparticles (ZnO-NPs + Se-NPs) were more numerous and larger, in addition to being richer in starch grains than in the control cells (Figure 9A,B,D,E). Moreover, large plastoglobules were observed in the control plants (Figure 9B). The chloroplasts in the plants that were treated with AMF and sprayed with a mixture of nanoparticles (ZnO-NPs + Se-NPs) were enlarged and had thicker grana that contained multiple layers of thylakoids (stacked grana thylakoids), as well as non-stacked stroma thylakoids when compared to the control plants (Figure 10A,B).

## 3. Discussion

Cold winter temperatures can negatively affect the reproductive stage, productivity, and quality of chili plants [1,2]. Based on our observations, AMF colonization and spraying with ZnO-NPs or Se-NPs have the potential to enhance cold-stress tolerance in crop plants. Plant growth characteristics are strong indicators of abiotic stress. In our study, we observed that the growth characteristics of chili plants significantly decreased under cold stress (Table 1). These findings are consistent with those reported by the authors of [26]. However, AMF inoculation significantly increased the growth characteristics of chili plants in both seasons. This demonstrates the role of AMF colonization in chili plants, even when they are exposed to low optimum temperatures [27]. These increases in plant growth parameters have been linked to more efficient micro and macronutrient uptake [27]. These results are consistent with those obtained in [28]. Under cold stress, the spraying of nanoparticles was associated with significantly higher plant growth parameters in comparison with the control treatment, indicating that ZnO-NPs and Se-NPs enhanced photosynthetic activity and antioxidant enzyme levels [29,30]. In addition, plants colonized with AMF and sprayed with (ZnO-NPs + Se-NPs) flowered earlier than control plants in both seasons (Figure 1). This is a highly desirable trait because it can directly influence market product prices. Our findings are strongly supported by those in [31]. 

The findings also illustrated that suboptimal temperatures decreased the marketable yield and its components (Figure 3A–D). Cold stress reduces productivity by slowing CO_2_ fixation and fruit photosynthetic partitioning [31]. These results are consistent with those previously reported [26,32]. In particular, AMF colonization combined with ZnO-NPs and Se-NPs spraying yielded drastically higher fruit weight, fruit number, and total marketable fruit yield in both seasons. Our observations are strongly supported by those of [33], who observed that AMF enhanced the productivity of snap bean crops. The positive impact of AMF on productivity could be owing to the production of glomalin (also known as glomalin-related soil protein (GRSP) or glycoprotein glomalin) released in the soil as an external secretion of AMF symbiosis, which has been proven to improve soil aggregate stability, soil water potential, and productivity. Moreover, the results demonstrated that AMF colonization combined with (ZnO-NPs + Se-NPs) spraying increased the total yield per plant by 95.8% and 94.7% in the 2021 and 2022 seasons, respectively. These increases could be attributed to the combination of nanoparticles and AMF, which have a synergistic effect against cold stress. Zinc oxide nanoparticles (ZnO-NPs) increased the productivity of wheat plants [34]. Zinc plays an important role in hormone metabolism regulation by altering auxin levels via tryptophan production and promoting cell division and expansion [35]. Furthermore, Se-NPs improved the total yield of groundnut cultivars [30]. 

The highest values of chlorophyll content, photosynthesis, transpiration rate, and stomatal conductance were recorded for AMF combined with nanoparticles mixture (ZnO-NPs + Se-NPs) in both seasons (Figure 2A–D). The observed impact of AMF on physiological features may be attributable to its contribution to decreasing N_2_O emissions by increasing N uptake, therefore improving photosynthesis and potassium uptake [34]. Zn is required for the activities of enzymes [23,24]. These findings are consistent with those reported by the authors of [34]. Generally, metallic nanoparticles have active effects on photosynthetic efficiency, which increases light absorption by chlorophyll [36]. 

According to the results, the fruit quality traits changed due to cold stress. Similar results were obtained for tomatoes by the authors of [32]. Chili fruits harvested from AMF with nanoparticles mixture (ZnO-NPs + Se-NPs) recorded high values of single-fruit weight, fruit diameter, fruit length, ascorbic acid, and capsaicin content in both seasons compared to the control (Table 2). ZnO-NP application enhanced the quality and nutrient content of harvested bell pepper fruits [37].

Under cold stress, the control plants displayed the lowest content of nutrients, including N, K, P, Ca, Fe, Mn, Zn, and Se, in both seasons (Table 3 and Table 4). In contrast, the results obtained from AMF combined with a nanoparticle mixture (ZnO-NPs + Se-NPs) showed better absorption of minerals under suboptimal temperatures in both seasons. AMF may exert its effect on the mineral content in plants by strigolactone production, revealed to be hyphal branching and host detection signals for AMF [38], which play an essential role in responses to abiotic stress conditions by improving growth and nutrient absorption. Se-NPs and ZnO-NPs exhibited remarkable nutrient content in the leaves of cowpea [39].

Plants have enzymatic defense systems to purify free radicals under cold stress. Changes in antioxidant enzyme levels have been associated with cold-stress reactions [32]. AMF combined with ZnO-NPs + Se-NPs improved the POD enzyme content in chili leaves in both seasons. These results are consistent with those reported by the authors [34]. AMF protects plants from cold stress and increases antioxidant enzyme levels [40]. ZnO-NPs cause changes in secondary chemicals relevant to plant defense against abiotic stress factors [41]. Se-NP treatment increased auxin production, therefore enhancing root architectural modifications in tomatoes [42]. GDH may have a special physiological role in the release of large quantities of ammonium in plants under cold stress [43]. These data indicated that AMF combined with ZnO-NPs + Se-NPs significantly improved the activity of the nitrogen metabolism enzyme (GDH) under cold stress. AMF improved N uptake and photosynthetic activity in chili leaves but led to a reduction in N_2_O emissions in plant leaves [44]. Zn increased the synthesis of proteins as cofactors in specific enzymes (DNA and RNA), chlorophyll, carbohydrates, lipids, and hormones [45]. Spraying of ZnO-NPs + Se-NPs stimulated plant cells and boosted cell metabolic activity [39,41], therefore improving cold tolerance in chili plants. 

ABA reactions control stomatal conductance and other physiological functions [45]. The results indicated that the lowest ABA content was registered in the chili plants colonized with AMF and sprayed with a nanoparticle mixture (ZnO-NPs + Se-NPs) in both seasons (Figure 4B). AMF inoculation affected stomatal function via abscisic acid modulate auxin concentration via tryptophan biosynthesis [45]. AMF combined with a nanoparticle mixture (ZnO-NPs + Se-NPs) decreased the MDA content and the H_2_O_2_ content in chili leaves compared to the control in the 2021 and 2022 seasons, respectively. Similar outcomes for wheat plants were reported in [34,46]. AMF increases antioxidant enzyme (POD), which eliminates ROS, therefore protecting plants from oxidative damage or membrane stabilization [43,45]. Zinc is a cofactor for numerous enzymes, allowing the antioxidant defense system under cold stress [45]. Selenium dismutation of O^2−^ and OH^−^ into H_2_O_2_ is followed by H_2_O_2_ scavenging by CAT antioxidant enzyme [47]. 

SDS-PAGE analysis demonstrated that ZnO-NPs, Se-NPs, AMF + ZnO-NPs + Se-NPs, and AMF + ZnO-NPs provided plants with a defense mechanism against cold stress compared to the control. Similar findings in wheat plants were reported by [34], who observed that AMF + ZnO-NPs enhanced the synthesis of new and distinctive bands in antioxidant enzymes as a stress-resistance strategy for wheat plants. Hence, the synthesis of unique bands is caused by several structural modifications of DNA that initiate variations in amino acids, resulting in protein formation [48]. In this regard, the authors of [49] hypothesized that an enzyme called Rubisco activase would be associated with a band with a molecular weight of 51 kDa. Furthermore, according to [50,51], under salinity stress, flax and sunflower plants express a protein with a molecular weight of 26 kDa to help their survival.

The combination of AMF inoculation and ZnO-NPs + Se-NPs as a foliar spray achieved an enhancement in most anatomical characteristics. The optimistic effect of AMF on the anatomical properties may be due to the vital role of AMF in mitigating crop abiotic stress by adjusting good root morphology using the extra-radical mycelium and improving the absorption of nutrients and water in the soil [52]. These findings are consistent with those reported by the authors of [53], who found that Zn and Se are crucial for promoting the chlorophyll scale and relieving all harmful outcomes caused by abiotic stress. In addition, Se-NPs improved the vascular bundle dimensions and thickness of mesophyll, xylem, and phloem, as well as the thickness of the lower and upper epidermis of Date Palm plants under salinity stress [54]. However, all anatomical characteristics decreased in the control plants. These results are in agreement with those reported by [55], who found that the cells of the upper and lower epidermis and mesophyll of wheat leaf plants that were not subjected to low temperature were closely coordinated, whereas the mesophyll cells of the plants exposed to low temperatures (0 °C at the elongation stage and 10 °C at tillering) decreased with increasing intercellular temperature.

Furthermore, AMF and a mixture of nanoparticles (ZnO-NPs + Se-NPs) increased the number of chloroplasts and improved the thickness of grana thylakoids. The stacked grana thylakoids in chili plants inoculated with AMF and sprayed with (ZnO-NPs + Se-NPs) were accompanied by the accumulation of starch granules, which indicates an increase in photosynthesis activity, as starch is the energy storage of the cells and is a product of photosynthesis. This agrees with the results obtained in [52] for peanuts and [56] for *Cucumis melo* L. In contrast, the number and size of plastoglobules within chloroplasts increased in the control plants. This agrees with the findings of [57], who observed that plastoglobules increased under cold stress because of their role in the active channeling of lipid molecules and lipid breakdown products.

## 4. Materials and Methods

### 4.1. The Experimental Location and Plant Materials

**Figure 11:**
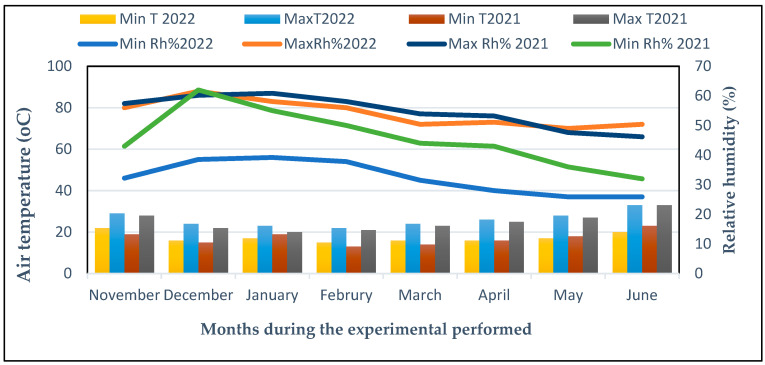
Climatic data for every month during the 2021 and 2022 seasons in The Experimental Field at Cairo University in Giza, Egypt. Minimum relative humidity (Min Rh%), minimum air temperature (Min T (°C)), and maximum air temperature (Max T, (°C)), maximum relative humidity (Max Rh%).

Two field trials were successfully performed throughout two winter seasons (December to June) in 2021 and 2022 at the Experimental Field, Vegetable Crops Department, Faculty of Agriculture, Cairo University, Giza, Egypt (30°1′10″ North latitude and 30°11′5″ East longitude). The climatic conditions at the growing location are shown in Figure 11. The chili (*C. annuum* L. Sina F1) cultivar was used as the experimental model in this study. The seeds of the chili pepper (Sina F1) cultivar were purchased from NEW Star Four Co., El Weili, Egypt. *Arbuscular mycorrhiza* fungus was produced by the Soil Department, Agricultural Research Centre, Ministry of Agriculture, Giza, Egypt. The AM fungal (*Glomus mosseae*) inoculum, containing hyphae and culture substrates, including spores (nearly 70 g^−1^), was dried in air.

### 4.2. Mycorrhizal Inoculums and Growth Conditions

Before sowing, the seeds of chili (cv. Sina F1) were sterilized in sodium hypochlorite at 5% for 15 min and then rinsed with distilled water. On 1 November 2021 and 2nd 2022, seeds of chili plants were germinated in seedling trays containing 10 cm^3^ cells filled with a mix of peat moss and vermiculite (1:1 v:v). Furthermore, half of the seedling trays received the AM fungi *(G. mosseae*) at a rate of 10 g inoculum per sowing cell, whereas the other half received non-AMF inoculum. The mycorrhizal inoculums were placed at 3–5 cm depth in compartments of trays directly before the growth of the chili seedlings to promote fungal inoculation of plant roots. The experimental trays were set up in a nursery with a relative humidity of 50–60%, an average temperature of 18–20 °C (day/night), with a 13/11 h (light/night) photoperiod, the photon flux density was in the range of 1100 µmol m^−2^ s^−2^.

### 4.3. Field Experiments

Uniform chili pepper seedlings (30 days old) were carefully planted in an open field on 1 December 2021 and 2nd 2022 seasons at the Eastern Field of Vegetable Crops Department, Faculty of Agriculture, Cairo University, Giza, Egypt. The experimental plot area was 16 m^2^ (8 m long and 2 m wide). The space between rows was 50 cm, and each transplant had a space of 30 cm. There were 48 plants in each replicate (six plants per treatment). Control chili seedlings (untreated seedlings) were grown under the same conditions. The application of Se-NPs or ZnO-NPs (10 mg/L) was performed three times. The first application was 10 days after transplanting (11 December 2021 and 13 December 2022), followed by two foliar sprays at intervals of 10 days. The experimental design included eight treatments: (T1) control (untreated plants), (T2) Se-NPs, (T3) ZnO-NPs, (T4) (ZnO-NPs + Se-NPs), (T5) AMF, (T6) AMF + Se-NPs, (T7) AMF + ZnO-NPs, (T8) AMF + (ZnO-NPs + Se-NPs). Six replicates were used for the treatments in a randomized complete block design (RCBD). Agricultural practices and soil preparation were implemented by the recommendations of the Egyptian Ministry of Agriculture. The physical characteristics of the experimental soils are presented in Table 7. 

### 4.4. Preparation of Nanoparticles

**Figure 12:**
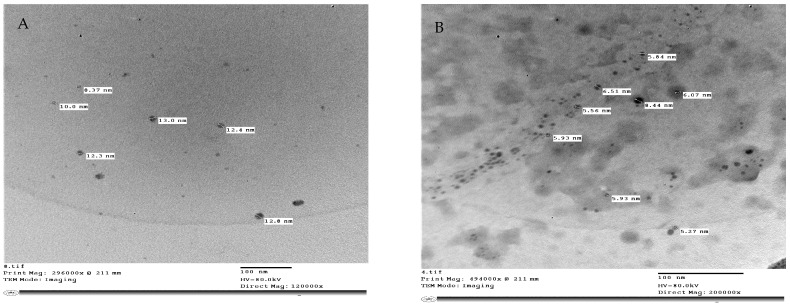
Transmission Electron Microscope (TEM) of (**A**) Zinc Oxide nanoparticles size from 8.37 to 12.8 nm, (**B**) selenium nanoparticles size from 5.27 to 6.51 nm. Scale bars = 100 nm.

Zinc oxide nanoparticles (ZnO-NPs) were formed from zinc chloride (General Purpose Reagent, minimum 98.5%, Sigma-Aldrich, Munich, Germany). Zink oxide nanoparticles (ZnO-NPs) (Figure 12A) were prepared using the method described in [58]. Zinc Oxide nanoparticles were synthesized from an aqueous solution containing zinc chloride. NaOH was gradually introduced at a molar ratio of 1:2 over 8 h. The obtained precipitate was thoroughly filtered and washed with ionized water in a mixture of water and toluene using a high-speed stirrer, followed by additional washing with only ionized water for 3 h. The precipitate was dried in an oven at 100 °C. Selenium nanoparticles (Se-NPs) (Figure 12B) were formed according to a previously described protocol [59]. Se-NPs were created by the chemical reduction of sodium selenite by glutathione. In particular, 9 mL of double-distilled water in a sterile cabinet was mixed with 3 mL of 25 mM Na_2_SeO_3_. A total of 1 M NaOH was added to the reactant solution after mixing to increase the pH to that of the alkaline media. Se-NPs were generated as soon as NaOH was added, as shown by the color change in the reactant solution from clear white to clear red. The structure and sizes of (ZnO-NPs) and (Se-NPs) were studied using a transmission electron microscope Jeol (JEM—1400 TEM). In addition, TEM imaging was performed in the TEM laboratory at the Research Park (CURP), Faculty of Agriculture, Cairo University, Cairo, Egypt.

### 4.5. Data Recorded 

#### 4.5.1. Plant Growth, Flowering, and Yield

Ten chili pepper plants were chosen from each treatment 60 days after transplanting to measure the plant height (cm) and the average number of leaves. In addition, a laser area meter (CI-202, Cedarhurst, NY, USA) was used to determine the leaf area (cm^2^). To determine plant biomass, each fresh sample was weighed and dried at 70 °C in an oven until it reached a constant weight. Days to flowering following plant transplanting were estimated from second internode flowers, and the number of flowers was counted when 50% of the chili plants flowered; during harvest, chili fruits were harvested when they were marketable in size (fruit with no obvious flaws and brilliant green color as described by [28]. At each harvest, the fresh fruit weight per plant (g) and fruit number per plant were estimated, and the total yield per plant (kg) and total yield per square meter (m^2^) were estimated after the fourth harvest.

#### 4.5.2. Photosynthetic Parameters and Chlorophyll Content of Chili Plants

Photosynthetic parameters (transpiration rate, photosynthesis rate, stomatal conductance, and intercellular CO_2_) were essayed using an infrared gas analyzer (LICOR 6400 Portable Photosynthesis System; IRGA, Licor Inc., Lincoln, NE, USA). To assess photosynthetic measurements, the fifth leaf of the chili plant was selected from each treatment 60 days after transplantation. Photosynthetic traits were evaluated between 10:00 a.m. and 1:30 p.m. with a light intensity of 1200 mol m^−2^ s^−1^ and 80% RH. Chlorophyll content was assessed using a SPAD meter (SPAD-502, Konica Minolta Sensing, Inc., Osaka, Japan) using the method described by [60], using the fifth leaf from each treatment. 

#### 4.5.3. Fruit Quality Parameters

At each harvest, four marketable chili fruits from each treatment were chosen to evaluate fruit diameter and length. The fruit diameter (cm) was assessed as the widest point in both perpendicular directions, and the two measurements were averaged to determine the fruit diameter. The fruit length (cm) was estimated from the tip point to the calyx. The total capsaicin content in the chili fruit was measured using high-performance liquid chromatography (HPLC) according to [61]. The ascorbic acid concentration was determined using a 2, 4 dinitrophenylhydrazine substance and a spectrophotometer at 540 nm, according to [62].

#### 4.5.4. Mineral Content in Chili Leaves 

Nutrient elements were determined by drying chili leaf samples at 70 °C for 24 h in an electric oven using the method outlined in [63]. Concentrated sulfuric acid (5 mL) was added to the samples, and the mixture was heated for 10 min to wet digest 0.2 g of the plant material. Perchloric acid (0.5 mL) was then added, and the mixture was heated until a clear solution was produced [63]. The total nitrogen content of the dried chili leaves was evaluated using the modified micro-Kjeldahl method, according to the authors of [63]. The phosphorus content of the dried chili leaves was estimated using the chlororstannousmolybdo phosphoric blue color method in sulfuric acid, as previously described [64]. Potassium content was estimated using a flame photometer apparatus (CORNING M 410, Germany), the calcium content in chili leaves was determined according to [65], and micronutrient concentrations (Zn, Se, Mn, Cu, and Fe) in chili leaves were estimated using an atomic absorption spectrophotometer with air-acetylene and fuel (PyeUnicam, model SP-1900, USA) [65,66].

#### 4.5.5. Antioxidants Enzyme, Hormone, Lipid Peroxidation (MDA), Hydrogen Peroxide, and Nitrogen Metabolism Enzyme

The chili leaf samples were prepared using the method described in [67] to estimate peroxidase (POD)activity. Leaf tissue (0.5 g) was frozen in liquid nitrogen and used to extract peroxidase enzymes. The chili leaf samples were centrifuged at 3930× rpm for 20 min after being ground in 10 mL of extraction buffer (50 mM phosphate buffer, pH 7), which also contained 0.5 mM EDTA and 2% PVPP (*w*/*v*). Using a spectrophotometric method, the reaction mixture that included 450 mL of 25 mM guaiacol, 450 mL of 225 mM H_2_O_2_, and 100 mL of crude enzymes was used to produce the supernatant, which was then used to assess the peroxidase activity. The method described in [68] was employed to estimate the extraction of Abscisic acid (ABA) from chili leaves [68]. The homogenized freeze-dried materials were incorporated into a mixture that contained butylated hydroxytoluene and 80% *v*/*v* methanol. Malondialdehyde (MDA) levels in chili leaves were estimated using the thiobarbituric acid (TBA) method [69]. Hydrogen peroxide (H_2_O_2_) in chili leaves was evaluated using the trichloroacetic acid (TCA) method [70]. A total of 1 mL of 0.1% TCA was used to ground 0.1 g of the leaf. The homogenate was then centrifuged at 12,000× *g* rpm. The phosphate buffer (pH 7.0) and supernatant were combined in equal parts, and 1 mL of 1M potassium iodide was added. The absorbance of the reaction mixture was measured at 390 nm. The activity of glutamate dehydrogenase (GDH) was measured as described previously [71].

#### 4.5.6. Protein Electrophoresis SDS-PAGE 

Quick freeze-dried leaf samples (10 g) were extracted with 1 mL of protein buffer, maintained overnight in a freezer, vortexed for 5 min, and then centrifuged at 10,000× *g* rpm at 4 °C for 15 min. Electrophoretic analysis of proteins was performed using sodium dodecyl sulfate-polyacrylamide gel electrophoresis (SDS-PAGE). SDS-PAGE was performed as previously described [72]. Standard protein markers (20.7–320.23 kDa; Sigma-Aldrich) were used to measure the molecular weight of the isolated proteins. The protein bands were marked with Coomassie Brilliant Blue G-250 (Sigma–Aldrich). The molecular weight (Mwt) of each protein was related to standard markers using polyacrylamide gel plates, then photographed, scanned, and analyzed using Quantity One software (Version 4.6.2).

#### 4.5.7. Anatomical of Chili Leaves

The tested specimens, including the upper leaf developed on the third branch from the base of the main stem of chili plants, were selected during the second growing season of 2022, 10 days after the third foliar application. A sample of approximately 1 cm of the specimens was taken from each treatment, killed, and fixed in FAA solution (5 mL of glacial acetic, 10 mL of formalin, 35 mL of water, and 50 mL of ethyl alcohol 70%), for 48 h at least. The chosen sample was washed in ethyl alcohol at 30%, dehydrated in a normal ethanol and butyl alcohol chain, buried in paraffin wax with a melting point of 56 °C, sliced to a thickness of 15 μm, stained with crystal violet and erythrosin, clarified in xylene, and mounted in Canada balsam. Transverse sections were obtained using a Leica Microtome RM 2125, micrographed, and measured using a Leica Light Image Analysis System DM 750 at the Research Park (CURP), Faculty of Agriculture, Cairo University. The following parameters were recorded: the thickness of the midvein (μm), lamina (μm), palisade tissue (μm), spongy tissue (μm), upper and lower epidermis (μm), bundles dimension (μm), and mean vessels diameter (μm).

#### 4.5.8. Ultrastructure of Chili Leaves

Chili pepper leaf pieces at the age of 45 days from transplanting were processed for TEM by pre-fixing with 5% glutaraldehyde in 0.05 M phosphate buffer (pH 7.2) for 3 h, and post-fixed with 2% osmium tetroxide for 4 h, then dehydrated in ethanol alcohol, and mounted in epoxy resin. Semi-thin sections (500 nm) were obtained using a Leica Ultracut UCT Ultramicrotome, stained with toluidine blue. Ultra-thin sections were prepared at 100 nm thickness, stained with uranyl acetate and lead citrate in accordance with [73], and then examined using a transmission electron microscope Jeol (JEM—1400 TEM) at the candidate magnification at the TEM laboratory, Research Park (CURP), Faculty of Agriculture, Cairo University. Images were captured by CCD camera model AMT, an optronics camera with 1632 × 1632-pixel format as a side-mount configuration. This camera uses a 1394 firewire board for acquisition.

#### 4.5.9. Statistical Analysis

MSTATC software was utilized to analyze the results of two successive growing seasons [74]. The LSD test was also performed to determine whether a statistical difference (*p* ≤ 0.05) existed in the average values [75]. 

## 5. Conclusions

This study proves that cold stress can adversely affect the growth and productivity of chili plants. Nevertheless, according to SDS-PAGE analysis, the application of ZnO-NPs, Se-NPs, AMF + ZnO-NPs, and AMF + ZnO-NPs + Se-NPs provided plants with a defense mechanism against cold stress compared to the control. Chili plants inoculated with AMF combined with a mixture of nanoparticles (ZnO-NPs + Se-NPs) exhibited better transpiration rate, chlorophyll (SPAD), stomatal conductance, and photosynthetic rate, which subsequently led to higher vegetative growth, flowering, productivity, mineral components, antioxidant enzymes, and nitrogen metabolism enzymes. Conversely, the combination of AMF with a nanoparticle mixture (ZnO-NPs + Se-NPs) recorded the lowest content of ABA, MDA, and H_2_O_2_ in the first and second seasons. AMF combined with a nanoparticle mixture (ZnO-NPs + Se-NPs) is the best treatment, resulting in the highest number of chloroplasts and stacked grana thylakoids in chili plants. Finally, we recommend this scientific approach to farmers as a suitable strategy for reducing the harmful effects of cold stress on chili plants. 

## Figures and Tables

**Table 1:** Effect of inoculation with *Arbuscular mycorrhiza* fungus (AMF) and foliar applications of nanoparticles on the plant growth characteristics of chili plants during the 2021 and 2022 seasons.

Treatment	Plant Height (cm)	Number of Leaves	Plant Fresh Weight (g)	Plant Dry Weight (g)	Leaf Area (cm^2^)
2021 Season					
Control	50.3 ^h^	40.3 ^h^	101.3 ^f^	18.6 ^f^	51.7 ^h^
Se-NPs	59.0 ^g^	52.0 ^g^	156.0 ^e^	29.7 ^e^	55.3 ^g^
ZnO-NPs	67.6 ^f^	63.0 ^f^	207.3 ^d^	38.3 ^d^	58.7 ^f^
ZnO-NPs + Se-NPs	77.0 ^e^	75.0 ^e^	255.7 ^c^	48.0 ^c^	61.0 ^e^
AMF	84.3 ^d^	88.7 ^d^	303.3 ^b^	52.3 ^bc^	65.0 ^d^
AMF + Se-NPs	92.3 ^c^	102.3 ^c^	318.7 ^b^	58.7 ^b^	67.7 ^c^
AMF + ZnO-NPs	102.3 ^b^	117.7 ^b^	312.0 ^b^	58.0 ^b^	71.0 ^b^
AMF + ZnO-NPs + Se-NPs	115.7 ^a^	135.7 ^a^	388.3 ^a^	74.7 ^a^	80.0 ^a^
LSD value at 0.05:	3.1	2.4	22.59	7.4	1.6
2022 Season					
Control	49.03 ^g^	35.0 ^g^	93.3 ^h^	14.7 ^g^	54.7 ^h^
Se-NPs	58.1 ^f^	55.3 ^f^	142.7 ^g^	25.0 ^f^	58.3 ^g^
ZnO-NPs	66.4 ^e^	61.0 ^f^	166.4 ^f^	31.9 ^e^	61.7 ^f^
ZnO-NPs + Se-NPs	76.0 ^d^	73.0 ^e^	203.1 ^e^	40.7 ^d^	63.4 ^e^
AMF	88.1 ^c^	86.6 ^d^	244.1 ^d^	50.2 ^c^	67.3 ^d^
AMF + Se-NPs	90.8 ^c^	99.7 ^c^	282.5 ^c^	61.1 ^b^	69.9 ^c^
AMF + ZnO-NPs	100.2 ^b^	110.3 ^b^	299.5 ^b^	62.0 ^b^	73.3 ^b^
AMF + ZnO-NPs + Se-NPs	113.6 ^a^	129.3 ^a^	336.2 ^a^	72.7 ^a^	82.3 ^a^
LSD value at 0.05:	6.3	8.09	7.1	3.3	1.7

Values followed by different letters are significant according to the LSD test (*p* ≤ 0.05%).

**Table 2:** Effect of inoculation with *Arbuscular mycorrhiza* fungus (AMF) and foliar applications of nanoparticles on the quality parameters of chili fruits during the 2021 and 2022 seasons.

Treatment	Single-Fruit Weight (g)	Fruit Length (cm)	Fruit Diameter (cm)	Capsaicin (µg/g)	Ascorbic Acid (mg/100 g)
2021 Season					
Control	13.0 ^e^	8.0 ^g^	0.67 ^e^	133.3 ^h^	85.5 ^f^
Se-NPs	15.3 ^c^	11.7 ^f^	1.4 ^d^	136.7 ^f^	89.2 ^de^
ZnO-NPs	14.3 ^d^	10.5 ^f^	1.03 ^e^	134.6 ^g^	88.4 ^ef^
ZnO-NPs + Se-NPs	16.7 ^b^	13.1 ^e^	1.0 ^d^	137.7 ^e^	92.0 ^d^
AMF	14.33 ^d^	14.6 ^d^	2.9 ^b^	139.2 ^d^	100.2 ^c^
AMF + Se-NPs	15.6 ^c^	17.8 ^b^	3.9 ^a^	140.5 ^b^	115.4 ^b^
AMF + ZnO-NPs	17.0 ^b^	16.2 ^c^	2.5 ^c^	139.6 ^c^	113.1 ^b^
AMF + ZnO-NPs + Se-NPs	18.6 ^a^	21.70 ^a^	3.5 ^a^	142.9 ^a^	125.0 ^a^
LSD value at 0.05:	0.93	1.38	0.39	0.34	3.3
2022 Season					
Control	15.3 ^d^	8.7 ^f^	0.63 ^e^	134.0 ^h^	88.0 ^f^
Se-NPs	17.0 ^bc^	14.4 ^d^	1.30 ^d^	137.5 ^f^	91.7 ^de^
ZnO-NPs	16.7 ^c^	13.0 ^e^	1.20 ^d^	135.3 ^g^	90.9 ^de^
ZnO-NPs + Se-NPs	16.7 ^c^	15.0 ^d^	1.7 ^c^	138.4 ^e^	94.5 ^d^
AMF	17.3 ^bc^	15.5 ^cd^	1.60 ^c^	139.7 ^d^	103.7 ^c^
AMF + Se-NPs	17.6 ^bc^	17.9 ^b^	2.6 ^a^	141.0 ^b^	119.4 ^b^
AMF + ZnO-NPs	18.0 ^b^	16.2 ^c^	2.1 ^b^	140.1 ^c^	117.1 ^b^
AMF + ZnO-NPs + Se-NPs	19.6 ^a^	21.0 ^a^	2.7 ^a^	143.3 ^a^	129.0 ^a^
LSD value at 0.05:	1.26	1.1	0.26	0.27	3.41

Values followed by different letters are significant according to the LSD test (*p* ≤ 0.05%).

**Table 3:** Effect of inoculation with *Arbuscular mycorrhiza* fungus (AMF) and foliar applications of nanoparticles on the macronutrient content of chili leaves during the 2021 and 2022 seasons.

Treatment	N%	P%	K%	Ca%
2021 Season				
Control	2.5 ^f^	0.08 ^d^	2.1 ^d^	0.6 ^d^
Se-NPs	3.0 ^e^	0.26 ^c^	3.2 ^c^	1.1 ^c^
ZnO-NPs	2.9 ^e^	0.27 ^bc^	3.1 ^c^	1.2 ^bc^
ZnO-NPs + Se-NPs	3.2 ^d^	0.31 ^bc^	3.4 ^bc^	1.3 ^bc^
AMF	3.3 ^c^	0.35 ^bc^	3.6 ^bc^	1.5 ^bc^
AMF + Se-NPs	3.4 ^c^	0.38 ^b^	3.7 ^b^	1.4 ^bc^
AMF + ZnO-NPs	3.7 ^b^	0.37 ^b^	3.8 ^b^	1.6 ^b^
AMF + ZnO-NPs + Se-NPs	3.9 ^a^	0.70 ^a^	4.4 ^a^	1.9 ^a^
LSD value at 0.05:	0.14	0.11	0.46	0.35
2022 Season				
Control	2.3 ^f^	0.12 ^h^	1.9	0.5 ^h^
Se-NPs	3.2 ^e^	0.35 ^f^	2.9 ^d^	0.9 ^g^
ZnO-NPs	3.3 ^e^	0.28 ^g^	2.9 ^cd^	1.2 ^f^
ZnO-NPs + Se-NPs	3.4 ^d^	0.43 ^e^	3.5 ^bc^	1.53 ^e^
AMF	3.5 ^cd^	0.50 ^d^	3.8 ^b^	1.8 ^d^
AMF + Se-NPs	3.6 ^c^	0.64 ^b^	3.9 ^b^	2.15 ^c^
AMF + ZnO-NPs	3.9 ^b^	0.58 ^c^	4.0 ^b^	2.4 ^b^
AMF + ZnO-NPs + Se-NPs	4.2 ^a^	0.84 ^a^	4.7 ^a^	2.7 ^a^
LSD value at 0.05:	0.44	0.055	0.61	0.11

Values followed by different letters are significant according to the LSD test (*p* ≤ 0.05%).

**Table 4:** Effect of inoculation with *Arbuscular mycorrhiza* fungus (AMF) and foliar applications of nanoparticles on the micronutrient content of chili leaves during the 2021 and 2022 seasons.

Treatment	Fe (ppm)	Mn (ppm)	Cu (ppm)	Zn (ppm)	Se (ppm)
2021 Season					
Control	56.2 ^h^	12.7 ^h^	2.1 ^g^	32.9 ^f^	0.5 ^g^
Se-NPs	60.1 ^g^	20.9 ^f^	2.5 ^f^	30.1 ^g^	1.4 ^cd^
ZnO-NPs	62.9 ^f^	18.1 ^g^	2.5 ^f^	37.6 ^d^	0.9 ^f^
ZnO-NPs + Se-NPs	65.1 ^e^	23.1 ^e^	2.7 ^e^	40.5 ^c^	1.6 ^bc^
AMF	67.6 ^d^	25.6 ^d^	2.9 ^d^	23.7 ^h^	1.3 ^de^
AMF + Se-NPs	70.5 ^c^	30.8 ^b^	3.04 ^c^	35.1 ^e^	1.7 ^b^
AMF + ZnO-NPs	72.8 ^b^	28.5 ^c^	3.3 ^b^	42.8 ^b^	1.1 ^ef^
AMF + ZnO-NPs + Se-NPs	78.2 ^a^	35.9 ^a^	3.5 ^a^	48.2 ^a^	2.2 ^a^
LSD value at 0.05:	1.14	1.41	0.078	0.99	0.22
2022 Season					
Control	55.0 ^h^	14.3 ^h^	2.13 ^h^	22.1 ^g^	0.75 ^f^
Se-NPs	60.50 ^g^	23.3 ^f^	2.8 ^f^	30.5 ^f^	1.18 ^cd^
ZnO-NPs	63.33 ^f^	20.2 ^g^	2.5 ^g^	35.3 ^de^	0.94 ^e^
ZnO-NPs + Se-NPs	66.0 ^e^	25.5 ^e^	3.16 ^e^	40.7 ^c^	1.3 ^bc^
AMF	68.0 ^d^	27.9 ^d^	3.5 ^b^	37.7 ^d^	1.02 ^de^
AMF + Se-NPs	71.0 ^c^	33.2 ^b^	3.4 ^c^	33.3 ^e^	1.4 ^b^
AMF + ZnO-NPs	73.3 ^b^	30.8 ^c^	3.3 ^d^	43.8 ^b^	1.11 ^c–e^
AMF + ZnO-NPs + Se-NPs	78.9 ^a^	39.8 ^a^	3.9 ^a^	46.9 ^a^	1.7 ^a^
LSD value at 0.05:	1.113	1.66	0.055	2.5	0.17

Values followed by different letters are significant according to the LSD test (*p* ≤ 0.05%).

**Table 5:** The effect of AMF inoculation, nanoparticle foliar application, and their combined interactions on protein banding patterns of SDS-PAGE of pepper plants subjected to cold stress. T1: control, T2: Se-NPs, T3: ZnO-NPs, T4: AMF + ZnO-NPs + Se-NPs, T5: AMF + Se-NPs, T6: AMF + ZnO-NPs, T7: AMF, T8: ZnO-NPs + Se-NPs.

Mwt (kD)	T1	T2	T3	T4	T5	T6	T7	T8
320.23	-	+	+	+	+	+	+	-
228.02	-	+	+	+	+	+	+	+
133.56	+	+	+	+	+	+	-	+
61.96	+	+	+	+	+	+	+	+
38.82	+	+	+	+	-	+	+	+
28.09	-	+	+	+	+	+	+	+
20.74	+	+	+	+	+	+	+	+
Number of bands	7	7	4	7	6	7	6	7

Mwt: Molecular weight, +: Presence of band, -: absence of the band.

**Table 6:** Counts and measurements in micro-meters (µm) of certain histological characters in the transverse sections of the chili pepper leaf, treated with *Arbuscular mycorrhiza* fungus (AMF) and foliar applications of nanoparticles, 45 days from transplanting (Means of three sections from three specimens).

Treatments	Histological Aspects							
	Thickness of Midvein	Thickness of Upper Epiderm	Thickness of Lower Epiderm	Thickness of Lamina	Thickness of Palisade Tissue	Thickness of Spongy Tissue	Dimensions of Main Midvein Bundle	The Mean Diameter of the Vessel
Control	939.651	14.854	19.954	279.527	119.885	160.205	280.090	19.285
Se-NPs	1110.333	14.920	20.639	309.849	125.253	184.596	350.562	20.861
ZnO-NPs	1234.779	15.560	20.354	334.560	126.276	208.284	394.060	20.901
ZnO-NPs + Se-NPs	1255.086	17.208	21.758	463.622	194.225	269.397	286.452	20.333
AMF	1200.086	16.450	21.070	341.252	126.057	215.195	382.586	20.900
AMF + Se-NPs	1230.381	15.845	21.465	320.655	120.221	200.434	422.038	21.347
AMF + ZnO-NPs	1245.915	17.107	22.164	397.821	140.370	257.451	466.477	21.445
AMF + ZnO-NPs + Se-NPs	1260.588	17.886	22.747	419.633	144.979	275.654	469.774	21.762

**Table 7:** Physical and chemical properties of the experimental soil location during the two cultivating seasons of 2021 and 2022.

Soil Parameters	2021	2022
Physical properties		
pH	7.45 ± 0.5	7.35 ± 0.3
Clay (%)	42.2 ± 1.3	40.5 ± 1.2
Silt (%)	27.2 ± 5.1	28.7 ± 2.2
Sand (%)	20.5 ± 3.1	22.8 ± 5.1
Soil texture	Clay loam	Clay loam
Chemical properties		
Soluble Cations (meq L^−1^)		
Mg^2+^	2.45 ± 0.4	1.98 ± 0.2
K^+^	0.30 ± 0.05	0.30 ± 0.03
Ca^2+^	3.44 ± 0.4	3.50 ± 0.5
Na^+^	1.61 ± 0.5	1.75 ± 0.4
Available phosphorus (mg L^−1^)	0.12 ± 0.03	0.1 ± 0.01
Total nitrogen (TN) (%)	0.19 ± 0.03	0.17 ± 0.01
Soluble Anions (meqL^−1^)		
HCO_3−_	0.70 ± 0.2	0.78 ± 0.1
Cl^−^	1.20 ± 0.3	1.50 ± 0.2
SO_4_^2−^	1.80 ± 0.5	1.35 ± 0.6

## Data Availability

Data are contained within the article.
